# Modified Decisional Conflict Scale for Primary Caregivers in Long-Term Care Facilities: Psychometric Validation Using Structural Equation Modeling

**DOI:** 10.3390/healthcare14121754

**Published:** 2026-06-18

**Authors:** Pai-Yueh Chen, Ying-Hua Chao, Yao-Ching Huang, Shi-Hao Huang, Ren-Jei Chung, Pi-Ching Yu, Bing-Long Wang, Hsiu-Ju Chang, Pi-Chen Chang, Shu-Min Huang, Chao-Hsi Huang

**Affiliations:** 1 School of Nursing, College of Nursing, Taipei Medical University, Taipei 11031, Taiwan; xuwuxiangchun92@gmail.com; 2 Department of Nursing, Yuanpei University of Medical Technology, Hsinchu 30015, Taiwan; chaoyh211@gmail.com; 3 Department of Medical Research, Tri-Service General Hospital, National Defense Medical University, Taipei 11490, Taiwan; ph870059@gmail.com; 4 School of Public Health, College of Public Health, National Defense Medical University, Taipei 11490, Taiwan; billwang1203@gmail.com; 5 Department of Chemical Engineering and Biotechnology, National Taipei University of Technology (Taipei Tech), Taipei 10608, Taiwan; hklu2361@gmail.com (S.-H.H.); rjchung@ntut.edu.tw (R.-J.C.); 6 Graduate Institute of Medical Sciences, College of Medicine, National Defense Medical University, Taipei 11490, Taiwan; yupichin1003@gmail.com; 7 Department of Health Administration, Asia University, Taichung 41354, Taiwan; 8 Department of Nursing, College of Nursing, National Yang Ming Chiao Tung University, Taipei 112304, Taiwan; 9 Department of Nursing, College of Nursing, Efficient Smart Care Research Center, National Yang Ming Chiao Tung University, Taipei 112304, Taiwan; 10 Department of Infection Control, Taipei Medical University Hospital, Taipei 11031, Taiwan; sharon0617@yahoo.com.tw; 11 Department of Computer Science and Information Engineering, National Ilan University, Ilan City 26047, Taiwan

**Keywords:** decisional conflict, shared decision-making, long-term care facilities, primary caregivers, confirmatory factor analysis, structural equation modeling

## Abstract

**Background:** Caregivers of long-term care (LTC) residents often face decisional conflict during unplanned hospitalization decisions. This study aimed to adapt and psychometrically validate a modified Decisional Conflict Scale (DCS) for primary family caregivers. **Methods:** A cross-sectional survey was conducted among 205 caregivers in 20 LTC facilities. Exploratory and confirmatory factor analyses (EFA/CFA) were performed on randomly split subsamples, and structural equation modeling (SEM) examined associations among Decision Antecedents, Decision-Making Process, and Decisional Conflict. Bollen–Stine bootstrap procedures were applied to provide robust estimates under slight deviations from multivariate normality. Given the cross-sectional design and single-sample nature of this study, the SEM findings should be interpreted as theory-informed associations rather than causal effects. Longitudinal or intervention-based studies are needed to establish temporal ordering and determine whether improvements in caregiver readiness and decision-making processes lead to subsequent reductions in decisional conflict. **Results:** The modified DCS demonstrated strong internal consistency and a single-factor structure (*α* = 0.98, factor loadings 0.83–0.90). SEM indicated that Decision Antecedents and Decision-Making Process were significantly associated with Decisional Conflict (*R*^2^ = 0.68). The mediation analysis suggested that the Decision-Making Process partially mediated the relationship between Decision Antecedents and Decisional Conflict. **Conclusions:** The modified DCS appears to be a reliable and valid instrument for assessing decisional conflict among LTC caregivers. Findings highlight the importance of caregiver readiness, support, and communication in shaping decisional experiences. Given the cross-sectional design and single sample, results should be interpreted as associations rather than causal effects. Future research should replicate these findings in larger, diverse samples and explore short-form versions of the scale.

## 1. Introduction

Population ageing has become a major global public health challenge, driving the expansion of long-term care (LTC) services and increasing the proportion of older adults living with multimorbidity, frailty, and cognitive impairment [1,2,3]. Recent global demographic reports indicate that by 2030, 1 in 6 people in the world will be aged 60 years or over, and the global population aged 60 years and older is projected to increase from 1.1 billion in 2023 to 1.4 billion by 2030 [1,2]. Against this global backdrop, Taiwan is experiencing a particularly rapid demographic transition toward a super-aged society [2]. This shift has intensified the demand for LTC services, especially for residents with chronic functional dependence and complex care needs [1,2,3,4].

Residents of LTC facilities are especially vulnerable to acute clinical deterioration, including infections, falls, dehydration, altered consciousness, and exacerbations of chronic diseases, which may lead to unplanned hospitalization [4]. In the present study, unplanned hospitalization refers to urgent but not necessarily immediately life-threatening situations in which transfer to an acute care hospital is considered because of a sudden change in the resident’s health condition. These decisions often arise under time pressure and uncertainty, particularly when residents have impaired decisional capacity or communication limitations [5,6,7].

This study uses the term primary family caregivers to refer specifically to family members who serve as the principal surrogate decision-makers for LTC residents; after first mention, the abbreviated term primary caregivers is used throughout. This definition distinguishes them from formal or professional caregivers employed by LTC institutions. In LTC settings, decisions regarding unplanned hospitalization are commonly made through a shared process in which healthcare professionals provide clinical assessment, explain treatment options and risks, and offer recommendations, whereas primary family caregivers contribute knowledge of the resident’s previously expressed wishes, values, and care goals [5,6,7]. Because many LTC residents have frailty, cognitive decline, or communication limitations, caregivers often function as key participants or surrogate decision-makers during urgent care transitions. Their decisional experiences may therefore directly influence communication quality, treatment alignment with care goals, and the overall consistency of shared decision-making.

For primary caregivers, decisions regarding unplanned hospitalization are often emotionally demanding and ethically complex. They must weigh the resident’s clinical condition, probable prognosis, perceived quality of life, and previously expressed wishes while also responding to family expectations and professional recommendations [5,6,7]. Previous studies have shown that caregivers involved in healthcare decision-making often experience uncertainty, emotional burden, and distress, particularly when adequate information and support are lacking [5,6,7,8]. Such uncertainty is closely related to decisional conflict, which refers to personal uncertainty about which course of action to take when choices involve risk, regret, or challenges to personal values [8].

Shared decision-making (SDM) is an important component of person-centered care, particularly when healthcare decisions involve uncertainty, risk, or preference-sensitive options [9,10,11,12,13]. In LTC settings, SDM is especially relevant because residents often have impaired decisional capacity and family caregivers frequently participate in decisions regarding hospitalization and ongoing care [14,15]. However, despite its recognized importance, the implementation and measurement of SDM in LTC remain limited, particularly in relation to surrogate family decision-makers [14,15].

The Decisional Conflict Scale (DCS), developed by O’Connor, is one of the most widely used instruments for assessing uncertainty in healthcare decision-making [8,16]. Although the original DCS has demonstrated strong psychometric performance across a range of clinical contexts, it was primarily designed for individuals making decisions about their own care [8,16]. Other instruments related to shared decision-making mainly assess communication quality, participation, or decision-making preferences rather than decisional conflict itself [17,18]. As a result, these tools capture related but distinct constructs and may not adequately reflect the decisional experiences of family caregivers acting as surrogate decision-makers.

These limitations are particularly important in LTC settings, where decisions are often made on behalf of frail or cognitively impaired residents and are shaped by ongoing caregiving responsibility, family dynamics, and cultural expectations [14,15,19]. This issue is especially relevant in Taiwan, where healthcare decisions are often shaped not only by individual preferences but also by filial responsibility, family consensus, and deference to professional authority [19]. Therefore, existing decisional conflict and shared decision-making instruments may not fully capture the relational, contextual, and culturally embedded nature of caregiver decision-making in LTC settings.

Although decisional conflict and shared decision-making have been widely studied in general medical settings, few validated instruments specifically assess decisional conflict among primary caregivers making surrogate decisions for LTC residents during unplanned hospitalization. Existing tools do not sufficiently capture the intersection of caregiver readiness, information adequacy, support, communication processes, and culturally embedded family decision-making in LTC settings. Therefore, a context-sensitive and psychometrically sound instrument is needed to assess decisional conflict in this population.

Accordingly, this study aimed to adapt and psychometrically evaluate a modified DCS for primary caregivers involved in shared decision-making regarding unplanned hospitalization in LTC settings, assess its reliability and validity, and examine the hypothesized associations among decision antecedents, the decision-making process, and decisional conflict [20,21,22]. The purpose of this study was not to redefine decisional conflict as a fundamentally new construct, but rather to examine whether the core concept of decisional conflict, originally developed for individuals making healthcare decisions about their own care, could be meaningfully adapted to a caregiver-mediated surrogate decision-making context in LTC settings.

This issue may be particularly relevant in Taiwan, where healthcare decisions are often embedded within family-centered norms, filial responsibility, family consensus, and deference to professional authority. However, in the present study, these cultural factors were considered as contextual background rather than directly measured constructs.

The novelty of the present study lies in adapting and validating the Decisional Conflict Scale for primary family caregivers who serve as surrogate decision-makers for long-term care residents during unplanned hospitalization decisions. Unlike previous DCS applications that primarily focused on individuals making decisions about their own healthcare, this study addresses a caregiver-mediated decision-making context characterized by resident frailty, cognitive impairment, urgent transfer decisions, family-centered deliberation, and culturally embedded caregiving responsibilities. Therefore, the modified DCS was designed to capture not only general decisional uncertainty but also caregiver readiness, information adequacy, perceived support, communication quality, and the relational context of surrogate decision-making in Taiwanese LTC settings.

## 2. Methods

### 2.1. Study Design and Participants

This study was a methodological study of instrument modification and psychometric validation, conducted using a cross-sectional survey design. The study aimed to adapt and evaluate a modified Decisional Conflict Scale for primary caregivers involved in shared decision-making regarding unplanned hospitalization in long-term care settings. The overall study process was informed by established recommendations for scale development, cross-cultural adaptation, and psychometric evaluation [20,21,22,23].

Participants were recruited through purposive sampling from 20 long-term care facilities located in northern, central, and eastern Taiwan between December 2023 and March 2024. Eligible participants were adults aged 20 years or older who served as primary family caregivers for residents in LTC facilities and who had participated in at least one major healthcare decision, such as hospitalization or palliative care, on behalf of the resident. Participants were also required to be able to read and complete a Chinese-language questionnaire. Individuals without relevant caregiving and decision-making experience or with self-reported cognitive impairment were excluded.

A total of 205 valid responses were collected. The sample size was considered acceptable for the present psychometric evaluation and structural equation modeling analysis based on commonly cited recommendations for latent-variable modeling and scale validation studies, which suggest that samples of approximately 200 cases are generally adequate for models with a moderate number of factors and observed variables [21,22]. Although larger samples would provide greater stability of parameter estimates and stronger external generalizability, the current sample size was considered sufficient for the planned analyses.

After data cleaning and verification of questionnaire completeness, the 205 valid responses were randomly divided into two independent subsamples for psychometric evaluation using a computer-generated random allocation procedure, resulting in 102 cases in the exploratory factor analysis (EFA) subsample and 103 cases in the confirmatory factor analysis (CFA) subsample. This approach was used to support independent exploration and confirmation of the factor structure and to improve the robustness of the psychometric validation process. Ethical approval was granted by the Institutional Review Board of Taipei Medical University (TMU-JIRB No. N202312045), and written informed consent was obtained from all participants prior to data collection [23,24,25,26,27,28,29,30].

### 2.2. Conceptual Framework

The conceptual framework of this study was developed as an integrated, theory-informed model to guide the adaptation and validation of the modified Decisional Conflict Scale for primary caregivers involved in shared decision-making regarding unplanned hospitalization in long-term care settings. The framework was primarily informed by O’Connor’s Decisional Conflict Theory [8], together with relevant literature on shared decision-making [9,10,11,12,13], caregiver-mediated decision-making in long-term care [5,6,7,14,15], and culturally embedded family decision-making in Taiwan [19]. Because no single existing model fully addresses surrogate decision-making by primary family caregivers in LTC settings, the present framework was constructed to reflect the relational and contextual nature of these decisions.

In the present framework, the Decision-Making Process was defined as the interactional and deliberative phase of hospitalization-related shared decision-making [9,10,11,12,13]. It included several theoretically grounded components: exchange of clinical information between healthcare professionals and caregivers, caregiver participation in discussion, clarification of the resident’s values and presumed preferences, deliberation regarding potential benefits and risks of hospitalization, and movement toward a shared or mutually understood decision. Therefore, this construct was not intended to serve as a generic proxy for shared decision-making, but rather to represent the specific communication and deliberation processes through which caregivers engage in hospitalization decisions for LTC residents.

In this framework, Decision Antecedents refer to preconditions that may influence caregivers’ readiness to engage in decision-making, including perceived information adequacy, emotional preparedness, and support from healthcare professionals and family members. Prior studies suggest that access to relevant information, decisional support, and clarity regarding care options are important foundations for effective participation in shared decision-making [8,11,13]. In long-term care settings, these antecedent conditions may be especially important because caregivers often face uncertainty, limited time, and emotionally difficult choices when residents experience acute health deterioration [5,6,7].

The Decision-Making Process refers to the extent to which decision-making is collaborative, communicative, and aligned with shared decision-making principles, including communication quality, participation, and value-based deliberation. Shared decision-making theory emphasizes that high-quality decisions arise from active communication, mutual exchange of information, and deliberation about values and care goals between healthcare professionals and patients or their surrogates [9,10]. Previous research has shown that better communication, greater participation, and clearer discussion of options are associated with more informed and value-congruent healthcare decisions [11,12,13,14,15].

Decisional Conflict refers to uncertainty and perceived difficulty in choosing among healthcare options, particularly when caregivers feel insufficiently informed, unsupported, or unclear about which decision best reflects the resident’s values and interests. According to O’Connor, decisional conflict arises when individuals experience uncertainty about which course of action to take, especially when they feel uninformed, unsupported, or unclear about personal values [8]. This concept is particularly relevant to primary caregivers in LTC settings, who often act as surrogate decision-makers for vulnerable residents and must balance medical information, family expectations, and the resident’s presumed wishes [5,6,7,19].

Based on these theoretical and empirical considerations, the framework proposed three hypothesized relationships. First, more favorable Decision Antecedents were expected to be positively associated with the Decision-Making Process because caregivers who feel better informed and supported are more likely to engage effectively in shared deliberation [8,11,13]. Second, a higher-quality Decision-Making Process was expected to be negatively associated with Decisional Conflict, as collaborative communication and value clarification may reduce uncertainty and emotional burden [8,11,12,13]. Third, Decision Antecedents were expected to have an additional direct association with Decisional Conflict since insufficient information and support may contribute to uncertainty even before or beyond the quality of the decision-making process itself [8].

**Figure 1:**
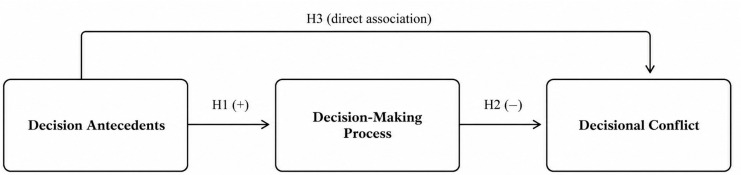
Hypothesized conceptual framework of the modified decisional conflict model for shared decision-making regarding unplanned hospitalization in long-term care settings.

Accordingly, Figure 1 presents the hypothesized conceptual framework of the study, showing the proposed associations among Decision Antecedents, the Decision-Making Process, and Decisional Conflict. Standardized path coefficients were not included in the conceptual framework and are reported only in the Section 3.

Based on the conceptual framework, the following hypotheses were proposed:

**H1:**  *Decision Antecedents are positively associated with the Decision-Making Process.*

**H2:**  *The Decision-Making Process is negatively associated with Decisional Conflict.*

**H3:**  *Decision Antecedents are directly associated with Decisional Conflict.*

The Decision Antecedents → Decision-Making Process → Decisional Conflict framework was developed as a contextual application of existing shared decision-making and decisional conflict theories rather than as a wholly original theoretical framework [8,9,10,11,12,13]. The model reflects the assumption that caregivers who feel better prepared, informed, and supported may be more able to participate in communication and collaborative deliberation, which in turn may be associated with lower decisional conflict [8,11,12,13]. Although this sequence is consistent with established SDM frameworks, the present study applies and empirically specifies these relationships in the context of primary family caregivers making or participating in unplanned hospitalization decisions for LTC residents [5,6,7,14,15,19]. The framework proposes that decision antecedents are positively associated with the decision-making process (H1), that the decision-making process is negatively associated with decisional conflict (H2), and that decision antecedents are directly associated with decisional conflict (H3).

Although the present framework focused on caregiver-reported decisional constructs, unplanned hospitalization decisions in LTC settings are also strongly shaped by resident-level clinical severity, including acute deterioration, frailty, comorbidity burden, cognitive impairment, and prognosis. These clinical factors were not modeled in the present study and should be incorporated into future expanded frameworks.

### 2.3. Instrument Development

The development of the modified Decisional Conflict Scale was guided by established recommendations for instrument development, scale validation, and cross-cultural adaptation [20,21,22,23]. Because the present instrument involved both adaptation of existing decisional conflict content and the addition of context-specific items for caregiver-mediated decision-making in long-term care settings, the development process combined principles of cross-cultural adaptation with de novo instrument development.

The modified instrument was based primarily on O’Connor’s original Decisional Conflict Scale (DCS) [8,16], which served as the core conceptual foundation for assessing uncertainty and perceived effectiveness in healthcare decision-making. To improve contextual relevance for primary caregivers involved in shared decision-making regarding unplanned hospitalization in LTC settings, additional content was informed by the shared decision-making literature and by related instruments addressing communication, participation, and decision preferences [24,25,26]. Accordingly, item development involved three sources: (1) items directly adapted from the original DCS, (2) items conceptually derived from related shared decision-making and decision-preference measures, and (3) newly generated items created to reflect the LTC caregiver context in Taiwan, including family-centered deliberation, emergency transfer decisions, and surrogate decision-making responsibilities.

The instrument development process was conducted in five steps. First, an initial item pool was generated on the basis of literature review, conceptual mapping, and review of relevant existing instruments. During this phase, the research team identified core domains considered theoretically relevant to caregiver decisional conflict in LTC settings, including decisional clarity, information adequacy, perceived support, values consideration, and confidence in the final decision. Items from the original DCS were reviewed for relevance and wording, while additional candidate items were drafted to address contextual elements not sufficiently covered by the original instrument.

Second, the preliminary item pool was reviewed by an expert panel consisting of specialists in long-term care, nursing, shared decision-making, and psychometrics. The panel evaluated each item in terms of conceptual relevance, clarity, cultural appropriateness, and suitability for caregiver-mediated decision-making in LTC settings. Items were retained, revised, merged, or removed according to these criteria, with particular attention paid to redundancy and conceptual overlap. Final decisions regarding item revision were made through expert consensus, and content validity evaluation was used to support item refinement.

Third, because the instrument included both adapted and contextually revised items for use in a Chinese-speaking population, cross-cultural adaptation procedures were undertaken. Forward translation and back-translation were performed independently by bilingual experts, followed by comparison and reconciliation of wording discrepancies. This process was intended not merely to achieve literal equivalence but also to ensure semantic, conceptual, and contextual appropriateness for primary family caregivers in Taiwan [22,27,28]. Although formal inter-rater agreement statistics such as kappa were not calculated, discrepancies in forward translation and back-translation were resolved through expert consensus to ensure semantic and conceptual equivalence.

Fourth, content validity was assessed through expert evaluation using relevance and clarity criteria. Each item was rated on a 4-point scale, and item-level and scale-level content validity indices were used to determine whether the retained items adequately represented the intended construct domain. Items that did not meet consensus standards were further revised before pilot testing.

Fifth, pilot testing was conducted with a small group of primary caregivers drawn from the target population to assess comprehensibility, acceptability, wording clarity, and response consistency. The pilot participants were similar in role to those included in the main study, as all had experience participating in healthcare-related decisions on behalf of LTC residents. Feedback from the pilot phase was reviewed qualitatively and used to identify ambiguous wording, improve readability, and refine item phrasing. Minor wording revisions were made before finalizing the instrument. The resulting final instrument comprised 16 items rated on a 5-point Likert scale. Reverse-coded items were retained to preserve conceptual consistency with the original Decisional Conflict Scale and to reduce acquiescence bias, with higher scores indicating lower decisional conflict after reverse coding where appropriate.

Thus, the final modified instrument represented a theory-informed and context-sensitive adaptation of the original DCS, developed through an integrated process that combined construct-based item development with cross-cultural adaptation procedures for use among primary caregivers in LTC settings.

### 2.4. Factor Analysis and Instrument Validation

The dataset was randomly split into two halves. One half (*n* = 102) was used for Exploratory Factor Analysis (EFA) to explore the underlying factor structure of the modified DCS, and the other half (*n* = 103) was used for Confirmatory Factor Analysis (CFA) to confirm the factor structure.

Exploratory Factor Analysis (EFA) was conducted on a randomly selected half of the sample (*n* = 102) to explore the underlying factor structure of the modified DCS. The Kaiser–Meyer–Olkin measure of sampling adequacy was 0.93, and Bartlett’s test of sphericity was significant (χ^2^ = 1423.57, *p* < 0.001), indicating suitability for factor analysis. Principal axis factoring with oblimin rotation was used. All 16 items loaded strongly on a single factor (loadings 0.83–0.90), explaining 68% of the total variance. The decision to retain a single-factor solution was supported by the eigenvalue > 1 criterion, scree plot inspection, and consistency with theoretical assumptions of overall decisional conflict.

Confirmatory Factor Analysis (CFA) was then conducted on the remaining half of the sample (*n* = 103) to test the hypothesized single-factor model. Model fit was evaluated using chi-square, CFI, TLI, RMSEA, and SRMR indices. Robust estimation methods (Bollen–Stine bootstrap) were applied to account for potential non-normality and to ensure stability of parameter estimates.

### 2.5. Data Collection Procedures

Data were collected using structured self-administered questionnaires distributed at participating LTC facilities. Trained research assistants followed a standardized protocol for participant approach, explanation of study objectives, informed consent procedures, questionnaire distribution, and collection. Their role was limited to procedural support, including answering general questions about study administration, and did not involve influencing participants’ responses or interpreting questionnaire content. This standardized approach was used to enhance consistency across study sites and minimize procedural bias. Questionnaires were completed anonymously and returned in sealed envelopes to protect confidentiality and support data integrity.

### 2.6. Data Analysis

All statistical analyses were conducted using IBM SPSS Statistics version 26 and AMOS version 24. Descriptive statistics were used to summarize participant characteristics and item distributions. Before factor and structural analyses were performed, assumptions relevant to covariance-based latent-variable modeling were examined. Item distributions were reviewed descriptively to assess normality, including skewness and kurtosis. Multicollinearity among observed indicators was also evaluated to ensure that the included variables were suitable for subsequent modeling.

#### 2.6.1. Reliability and Validity Testing

Internal consistency reliability was evaluated using Cronbach’s *α*, composite reliability (CR), and average variance extracted (AVE) [29]. Convergent validity was considered acceptable when CR was greater than 0.70 and AVE exceeded 0.50 [30]. Discriminant validity was assessed using the Fornell–Larcker criterion and the heterotrait–monotrait (HTMT) ratio, with values below 0.85 considered acceptable [31,32,33,34,35,36,37].

#### 2.6.2. Factor Structure

Although caregiver decision-making in LTC settings may involve multiple cognitive, emotional, ethical, and relational dimensions, the present CFA specified decisional conflict as a single latent construct for three reasons. First, the modified instrument was adapted from O’Connor’s DCS, which has commonly been used to assess an overall level of decisional conflict in healthcare decision-making. Second, the purpose of this initial validation study was to examine whether the adapted items formed a coherent general decisional conflict factor among primary caregivers involved in unplanned hospitalization decisions. Third, a unidimensional specification provides a parsimonious measurement model suitable for evaluating the overall psychometric performance of the modified scale. Therefore, the one-factor model was intended to represent overall decisional conflict rather than to imply that caregiver decision-making experiences are conceptually unidimensional. Given the theoretical foundation of the DCS, CFA was performed to test the hypothesized one-factor model. Maximum likelihood (ML) estimation was used. Because multivariate normality was not fully satisfied, the Bollen–Stine bootstrap procedure was applied to provide a more robust evaluation of overall model fit under non-normal data conditions [32]. This procedure was selected because the primary concern of the present study was the adequacy of global model fit in covariance-based latent-variable modeling, rather than only correction of individual parameter estimates.

The hypothesized one-factor model was retained on the basis of both statistical adequacy and conceptual coherence with the theoretical framework. Because the primary purpose of the CFA was to evaluate a theory-informed measurement structure, model retention was based on global fit indices and standardized factor loadings rather than data-driven specification alone.

Model fit was evaluated using multiple indices, including the chi-square to degrees-of-freedom ratio (χ^2^/df < 3), Goodness-of-Fit Index (GFI > 0.90), Adjusted Goodness-of-Fit Index (AGFI > 0.90), Root Mean Square Error of Approximation (RMSEA < 0.08), Standardized Root Mean Square Residual (SRMR < 0.08), Tucker–Lewis Index (TLI > 0.90), and Comparative Fit Index (CFI > 0.90) [33]. The hypothesized model was retained on the basis of both statistical adequacy and conceptual coherence with the theoretical framework.

#### 2.6.3. Structural Equation Modeling Procedures

Structural Equation Modeling (SEM) was conducted to examine associations among the study constructs, specified as Decision Antecedents → Decision-Making Process → Decisional Conflict. Latent constructs were defined according to the conceptual framework: Decision Antecedents reflected caregivers’ readiness and support prior to deliberation, Decision-Making Process captured communication quality, participation, and collaborative deliberation, and Decisional Conflict represented uncertainty and perceived difficulty in healthcare decision-making. Observed indicators for each construct are summarized in Supplementary Tables S1–S5.

Given cross-sectional data, directional paths should be interpreted as theory-informed associations rather than causal effects, and the model does not capture dynamic or recursive changes in decision-making over time. Bollen–Stine bootstrap procedures (*n* = 5000 resamples) were applied to provide robust parameter estimates and model fit under slight deviations from multivariate normality, which were observed in several variables. This approach is suitable for moderate sample sizes (*n* = 205). Future studies may compare bootstrap with robust maximum likelihood or alternative estimation methods to assess the stability of SEM results and the impact of non-normality.

## 3. Results

### 3.1. Participant Characteristics

A total of 205 valid questionnaires were collected from primary caregivers across 20 LTC facilities. Among them, 189 caregivers reported prior shared decision-making experience, whereas 16 reported no prior shared decision-making experience. To improve comparability, demographic characteristics for the two groups were presented using a standardized set of shared variables (Table 1).

### 3.2. Reliability and Validity Analysis of the Modified DCS

The modified DCS consisted of 16 items covering caregivers’ clarity, information sufficiency, perceived support, and confidence in making hospitalization decisions. Reliability and validity statistics are summarized in Table 2.

All standardized factor loadings ranged from 0.83 to 0.90, well above the acceptable threshold (≥0.50). Cronbach’s α coefficient was 0.98, and CR was 0.98, confirming excellent internal consistency. The average variance extracted (AVE) was 0.78, indicating strong convergent validity [34].

### 3.3. Confirmatory Factor Analysis (CFA)

**Figure 2:**
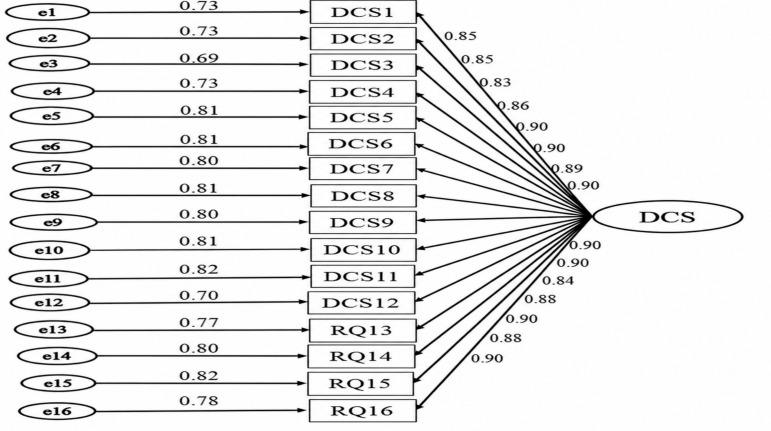
Confirmatory factor analysis of the modified Decisional Conflict Scale.

The CFA confirmed the factorial validity of the modified DCS (Figure 2), and model fit indices are summarized in Table 3. All path loadings were statistically significant (*p* < 0.001). The model demonstrated excellent overall fit: *χ*^2^ = 156.324, df = 104, *χ*^2^/df = 1.503, GFI = 0.966, AGFI = 0.961, RMSEA = 0.050, SRMR = 0.038, TLI = 0.987, and CFI = 0.988.

These results met all recommended criteria (*χ*^2^/df < 3, GFI > 0.90, RMSEA < 0.08, SRMR < 0.08, TLI > 0.90, CFI > 0.90) [35,36].

Figure 2 illustrates the standardized path loadings for all 16 items of the DCS model. All loadings ranged between 0.83 and 0.90, demonstrating strong relationships between observed indicators and the latent construct of decisional conflict. Measurement error variances were minimal, and covariance estimates between residuals remained non-significant.

### 3.4. Structural Equation Modeling (SEM)

Detailed results of the measurement model, item-to-construct allocation, and mediation analysis, including direct, indirect, and total effects with bootstrap confidence intervals, are provided in the Supplementary Materials (Tables S1–S4).

In the structural equation modeling (SEM), Decision Antecedents were represented by items reflecting caregivers’ informational readiness, perceived support, and preparedness for decision-making; Decision-Making Process was represented by items reflecting communication, participation, and collaborative deliberation during hospitalization decisions; and Decisional Conflict was represented by the 16-item modified DCS. A detailed summary of item allocation, factor composition, and mediation results is provided in the Supplementary Materials.

**Figure 3:**
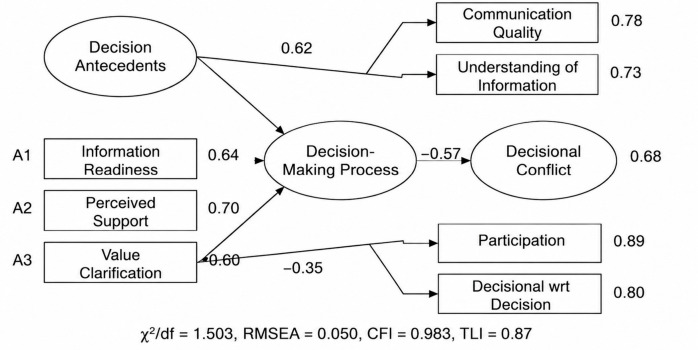
Structural equation model for Decision Antecedents, Decision-Making Process, and Decisional Conflict. Rectangles represent observed indicators, and ovals represent latent constructs. Solid arrows indicate estimated structural or measurement paths among constructs and indicators. The dashed arrow indicates the direct negative association between the Decision-Making Process and Decisional Conflict. Standardized coefficients are shown beside the corresponding paths. Model fit indices were as follows: χ^2^/df = 1.503, RMSEA = 0.050, CFI = 0.983, and TLI = 0.87.

The SEM examined the theoretical relationships among Decision Antecedents, Decision-Making Process, and Decisional Conflict (Figure 3).

Model results revealed that Decision Antecedents were positively associated with the Decision-Making Process (*β* = 0.62, *p* < 0.001), which in turn negatively predicted Decisional Conflict (*β* = −0.57, *p* < 0.001). The direct pathway from Decision Antecedents to Decisional Conflict remained significant (*β* = −0.35, *p* < 0.01), indicating partial mediation.

The overall model explained 68% of the variance (*R*^2^ = 0.68) in Decisional Conflict. Bootstrapping with 2000 resamples confirmed the significance of indirect effects (95% CI = −0.42 to −0.11), supporting the mediating role of the Decision-Making Process.

Figure 3 displays the standardized paths among latent variables. Decision Antecedents were positively associated with the Decision-Making Process (*β* = 0.62), which was subsequently associated with lower Decisional Conflict (*β* = −0.57). The direct association between Decision Antecedents and Decisional Conflict remained significant, supporting partial mediation.

### 3.5. Summary of Findings

In summary, the modified DCS exhibited:

- High reliability (α = 0.98);
- Excellent convergent and discriminant validity.
- Strong model fit in CFA and SEM analyses.
- Theoretical support was found for the view that caregivers’ readiness, information adequacy, and perceived support were associated with better decision-making processes and lower decisional conflict during unplanned hospitalization events in LTC settings.

## 4. Discussion

The findings should be interpreted within the Taiwanese LTC context in which the study was conducted. Caregiver-mediated decision-making may be shaped by family-centered norms, filial responsibility, family hierarchy, and deference to healthcare professionals. Therefore, the applicability of the modified DCS to other cultural or healthcare settings should not be assumed without further validation. The present findings provide initial psychometric evidence for use in Taiwanese LTC facilities and may be most relevant to similar family-centered care contexts.

### 4.1. Principal Findings

This study developed and validated a modified DCS specifically designed to assess the decisional experiences of primary caregivers involved in shared decision-making (SDM) regarding unplanned hospitalization in LTC facilities. Overall, the findings indicate that the modified DCS demonstrated strong psychometric performance and appears to be a valid and reliable instrument for use in this context. The scale showed excellent internal consistency, with Cronbach’s *α* = 0.98, composite reliability (CR) = 0.98, and average variance extracted (AVE) = 0.78, supporting both reliability and convergent validity [34]. In addition, all 16 items loaded strongly on a single latent construct, further supporting the internal coherence of the modified instrument [34,35,36,37,38,39,40].

At the same time, the very high internal consistency observed in this study should be interpreted with some caution. Although such findings support the reliability of the instrument, they may also suggest a degree of conceptual overlap among items. This possibility is particularly relevant given that all 16 items demonstrated high factor loadings on a single factor. Therefore, while the present results support the use of the full 16-item scale in this initial validation study, future research should examine whether a shorter and more efficient version could retain acceptable psychometric performance while improving feasibility for routine clinical use [21,22].

Although the present study was conducted in Taiwan, the cultural context should be interpreted primarily as an important background condition rather than as an empirically tested construct [19]. Family-centered decision-making, filial responsibility, family consensus, and deference to professional authority may shape how primary caregivers experience decisional conflict in LTC settings [19]. However, these cultural factors were not directly operationalized or measured as latent constructs in the present model. Therefore, the findings should not be interpreted as direct evidence of cultural mechanisms, but rather as initial evidence from a Taiwanese LTC context that requires further cultural measurement and validation.

The CFA supported a single-factor structure of the modified DCS, and the structural equation model further suggested that the Decision-Making Process played a significant mediating role in the relationship between Decision Antecedents and Decisional Conflict. These findings indicate that caregiver readiness, informational adequacy, and perceived support were meaningfully associated with decisional experiences when primary caregivers were required to make urgent healthcare decisions on behalf of LTC residents [40,41,42,43,44,45,46,47,48,49]. The CFA demonstrated acceptable to excellent model fit indices, including χ^2^/df = 1.503, GFI = 0.966, RMSEA = 0.050, and CFI = 0.988, all of which met recommended thresholds [37,38,39]. Decision Antecedents were positively associated with the Decision-Making Process (*β* = 0.62), indicating that caregiver preparedness, information adequacy, and perceived support were related to more effective engagement in SDM. The Decision-Making Process was negatively associated with Decisional Conflict (*β* = −0.57), suggesting that better communication, participation, and collaborative deliberation were associated with lower caregiver uncertainty and emotional burden during urgent hospitalization decisions [40,41,42,43,44,45,46,47,48,49]. In addition, the direct association between Decision Antecedents and Decisional Conflict remained significant (*β* = −0.35), indicating partial mediation and suggesting that pre-decisional conditions may influence caregiver conflict both directly and indirectly through the quality of the decision-making process.

Nevertheless, these structural findings should be interpreted cautiously. Although the SEM was specified in a directional manner on the basis of decisional conflict theory and prior SDM literature, the present study used a cross-sectional design. As a result, the observed relationships among Decision Antecedents, the Decision-Making Process, and Decisional Conflict do not establish temporal ordering or causality. Rather, the SEM findings support the plausibility of the proposed theoretical framework and indicate that the associations among these constructs are consistent with the hypothesized model [40,41,42,43,44,45,46,47,48,49]. Therefore, the directional paths should be understood as theory-informed and association-based rather than causal. Future longitudinal or intervention-based studies are needed to clarify temporal relationships and determine whether improvements in caregiver readiness and decision-making processes are followed by subsequent reductions in decisional conflict over time [21,22].

### 4.2. Comparison with Previous Studies

The present findings are broadly consistent with previous studies emphasizing the importance of information adequacy, decisional support, and participatory communication in reducing decisional conflict during healthcare decision-making [40,41,42]. Shared decision-making has been increasingly recognized as an essential component of high-quality, person-centered care, particularly when decisions involve uncertainty, risk, or preference-sensitive options [40,41]. Decision support interventions have also been shown to improve knowledge, clarify values, and reduce uncertainty among individuals facing treatment or screening decisions [42]. In this regard, the current findings support the view that decisional conflict among primary caregivers in long-term care settings is not an isolated phenomenon, but rather reflects broader patterns identified across healthcare decision-making research.

Our results further suggest that favorable decision antecedents, such as informational readiness, emotional preparedness, and perceived support, are positively associated with the quality of the decision-making process. This interpretation is consistent with prior studies indicating that shared decision-making is more likely to be effective when participants feel informed, supported, and prepared to engage in deliberation [43,44]. Previous research has shown that decision aids and communication strategies that facilitate discussion of options, benefits, harms, and patient values can strengthen participation and improve the quality of healthcare decisions [43,44]. Therefore, the positive association observed between Decision Antecedents and the Decision-Making Process in the present study is theoretically and empirically plausible.

The finding that a higher-quality decision-making process is associated with lower decisional conflict is also consistent with prior literature. Shared decision-making has been described not only as a communication model but also as a practical process through which uncertainty can be reduced by improving understanding, clarifying preferences, and supporting deliberation [45,46]. Previous studies have shown that when healthcare professionals actively engage patients or families in structured discussion and provide appropriate decisional support, the resulting decisions are often associated with greater confidence, less distress, and lower decisional conflict [46,47]. Our results extend these observations to the long-term care context, suggesting that communication quality and caregiver participation may play similarly important roles when family members act as surrogate decision-makers for vulnerable residents.

The present findings are also aligned with studies highlighting the relational and emotional burden of surrogate decision-making. Prior research has shown that family members making healthcare decisions on behalf of others often experience decisional burden, uncertainty, and emotional distress, particularly when the patient’s wishes are unclear, or the clinical situation is complex [48]. In long-term care settings, this burden may be intensified by frailty, dementia, progressive functional decline, and the need to reconcile medical recommendations with family interpretations of the resident’s best interests. In this respect, the current findings support the view that decisional conflict should be understood not only as an individual cognitive state but also as a relational and context-sensitive experience shaped by caregiving responsibility and communication processes.

Recent shared decision-making research has also emphasized that decisional outcomes are influenced by the structure of communication, the availability of support, and the quality of family–professional interaction rather than by individual preferences alone [49,50,51,52]. This perspective is particularly relevant to LTC settings, where hospitalization decisions are often made in time-sensitive and emotionally charged circumstances. Compared with many previous SDM studies that focused on patient-centered decision-making in outpatient or disease-specific contexts, the present study extends the literature by examining surrogate decision-making among primary caregivers in LTC settings through a process-oriented framework. In doing so, it contributes a more context-sensitive understanding of how pre-decisional conditions, communication processes, and decisional conflict are interconnected in caregiver-mediated healthcare decisions.

Taken together, these comparisons suggest that the present model is not merely another application of decisional conflict theory, but rather an extension of prior work into a clinically distinct context in which surrogate decision-making, family involvement, and culturally embedded care expectations are central. By linking Decision Antecedents, the Decision-Making Process, and Decisional Conflict within a single framework, the present study offers a more integrative account of caregiver decisional experiences in LTC settings and provides an empirical basis for future intervention and implementation research [40,41,42,43,44,45,46,47,48,49].

Recent evidence highlights determinants of shared decision-making and surrogate decision processes in long-term care and high-complexity care contexts [53,54,55,56,57]. For example, person-centered care environments and staff communication quality are associated with greater SDM engagement in LTC settings [53], and caregivers’ emotional burden, decision regret, and decision self-efficacy have been shown to influence surrogate decisional conflict and outcomes [55,56]. Scoping reviews further emphasize the complexity of including cognitively impaired care recipients and family involvement in SDM frameworks [54], and clinician–family communication patterns have been quantified in recent critical care goal-setting studies [57].

### 4.3. Theoretical Implications

The present study should be interpreted primarily as a context-sensitive adaptation of O’Connor’s Decisional Conflict Scale (DCS) for primary family caregivers in long-term care (LTC) settings, rather than a paradigm-level theoretical advancement [50,51]. Its contribution lies in empirically examining the feasibility, reliability, and validity of the modified DCS, and in providing initial evidence of associations among decision antecedents, decision-making processes, and overall decisional conflict in surrogate decision-making contexts. The Decision-Making Process construct was refined to capture theoretically meaningful components of shared decision-making, including information exchange, caregiver participation, clarification of the resident’s values and presumed wishes, discussion of risks and benefits, and collaborative deliberation [9,10,11,12,13,40,41]. This granularity highlights that decisional conflict may arise from inadequate information, insufficient participation, unclear values, or communication gaps, rather than from knowledge deficits alone [50,51]. Caregiver decisional conflict in LTC is inherently multidimensional, encompassing cognitive uncertainty, emotional distress, ethical burden, family responsibility, social expectations, and concern for the resident’s welfare [48,51]. The single-factor CFA model assessed overall decisional conflict but does not capture these subdomains. Similarly, the framework did not explicitly model ethical burden, moral distress, family hierarchy, or clinician–caregiver power imbalance, all of which may shape participation, support, and perceived responsibility in surrogate decision-making [19,48,51,52]. Future research should examine multidimensional, hierarchical, or bifactor models and consider these relational, ethical, and cultural factors. Overall, this study provides an applied extension of the DCS in a culturally and clinically distinct LTC context, offering a more nuanced understanding of how pre-decisional conditions and decision-making processes influence caregiver decisional conflict. The findings support the need for context-appropriate models of decisional burden, communication, and value-congruent care, and establish a foundation for future research on caregiver support, SDM interventions, and scale adaptation in LTC practice [50,51,52].

### 4.4. Practical and Clinical Implications

The present study also has important practical and clinical implications for LTC practice. The modified DCS provides healthcare professionals with a context-sensitive and psychometrically supported instrument for assessing decisional conflict among primary caregivers involved in unplanned hospitalization decisions. In LTC settings, such decisions often occur under time pressure and uncertainty, particularly when residents have frailty, dementia, or impaired communication ability. Under these circumstances, caregivers may experience uncertainty, emotional burden, and difficulty balancing medical recommendations with the resident’s presumed wishes and family expectations [53,54,55]. The modified instrument may therefore serve as a useful assessment tool for identifying caregivers who require additional information, emotional support, or structured communication before a final decision is made.

From a clinical perspective, the findings highlight several actionable targets for improving SDM in LTC settings. Because Decision Antecedents were positively associated with the Decision-Making Process, and the Decision-Making Process was negatively associated with Decisional Conflict, the results suggest that caregiver decisional experiences may be improved by strengthening preparedness, informational adequacy, and perceived support before and during hospitalization discussions. In practice, this means that clinicians may be able to reduce caregiver decisional conflict by providing timely explanations of the resident’s condition, clarifying possible options and likely outcomes, eliciting caregiver concerns and values, and ensuring that family members understand the rationale for transfer-related recommendations [53,54]. These actions may help caregivers feel more informed, more supported, and more capable of participating meaningfully in SDM.

The modified DCS may also be used at multiple points within routine care. First, it may be administered before or during acute transfer discussions to identify caregivers who are at risk of decisional distress or who appear insufficiently informed. Second, it may be used after family meetings or hospitalization decisions to evaluate whether the communication process effectively reduced conflict and improved confidence in the final decision. Third, it may serve as an outcome measure for SDM interventions, family conference models, staff communication training, or decision-support programs designed for LTC facilities [53,54,55]. In this respect, the instrument has value not only as a research measure but also as a potentially useful tool for clinical assessment and service evaluation.

In addition, the scale may support more structured interdisciplinary care. Nurses, case managers, social workers, physicians, and care coordinators often participate in discussions about hospitalization, prognosis, and goals of care in LTC settings. The modified DCS may help these professionals identify where decisional difficulty is arising—for example, whether caregivers feel uncertain because they lack information, feel unsupported by staff or relatives, or remain unclear about the resident’s values and preferences. By making decisional conflict more visible and assessable, the instrument may facilitate more targeted communication and more responsive caregiver support [53,54]. This may be particularly useful in situations involving repeated transfers, conflicting family opinions, or emotionally difficult choices regarding aggressive treatment versus comfort-focused care.

At the organizational level, the modified DCS may also contribute to quality improvement. LTC facilities could use aggregated caregiver responses to monitor decisional experiences across units or over time, identify communication gaps in urgent care transitions, and evaluate whether family-centered care initiatives are improving the quality of SDM. In this sense, the instrument may function not only as an individual-level screening tool but also as a system-level indicator of how effectively an institution supports caregiver participation in difficult healthcare decisions [53,54,55]. Such use may be especially valuable in settings seeking to improve ethical decision-making, reduce avoidable conflict, and promote more person- and family-centered transitions of care.

Overall, the practical contribution of this study is not merely that it modifies an existing scale, but that it provides a clinically relevant tool tailored to the realities of LTC decision-making. Its added value lies in helping clinicians recognize caregiver decisional burden more systematically, translate SDM principles into measurable practice, and support better communication during unplanned hospitalization decisions. Future implementation studies should examine how the modified DCS performs in routine LTC workflows and whether its use can enhance caregiver support, decision quality, and the overall consistency of family-centered SDM in practice [53,54,55].

The modified DCS should be interpreted as a potential assessment or screening tool for identifying caregiver decisional conflict, rather than as a clinical decision-support intervention by itself. The present study evaluated the psychometric properties of the modified instrument but did not test an intervention pathway or implementation mechanism. For clinical use, the scale would need to be embedded within a broader care process, such as structured family meetings, clinician–caregiver communication protocols, decision aid delivery, interdisciplinary care conferences, or caregiver support programs. In such a workflow, the modified DCS may help clinicians identify caregivers with high decisional conflict and guide targeted support, including clarification of clinical information, discussion of risks and benefits, value clarification, and emotional support. Future implementation studies are needed to determine whether incorporating the modified DCS into routine LTC practice improves communication quality, caregiver support, decisional conflict, or decision quality.

### 4.5. Limitations and Future Research

Several limitations should be acknowledged. First, participants were recruited through purposive sampling from 20 long-term care facilities in Taiwan. Therefore, the findings may not be generalizable to all LTC settings, other healthcare systems, or culturally different decision-making contexts. Although the total sample size of 205 participants was considered acceptable for an initial psychometric validation study, the random division of the sample into exploratory factor analysis and confirmatory factor analysis subsamples resulted in relatively small subgroup sample sizes. Specifically, the EFA was conducted with 102 participants and the CFA with 103 participants. This may have limited the stability of factor extraction, standardized factor loadings, model fit indices, and parameter estimates. Accordingly, the observed single-factor solution should be interpreted as preliminary evidence rather than definitive confirmation of the factor structure of the modified DCS. Future studies should validate the modified DCS in larger and independent samples to improve the stability, reproducibility, and generalizability of the factor solution.

Second, the present study used a cross-sectional design. Although the SEM paths were specified according to decisional conflict theory and shared decision-making literature, the data do not establish temporal ordering or causality. Therefore, the associations among Decision Antecedents, the Decision-Making Process, and Decisional Conflict should be interpreted as theory-informed associations rather than causal effects. Similarly, the observed mediation effect should not be interpreted as evidence of causal mediation. Longitudinal or intervention-based studies are needed to determine whether improvements in caregiver readiness, informational support, and communication processes lead to subsequent reductions in decisional conflict over time.

Third, all data were collected using self-reported questionnaires, which may have introduced recall bias, response bias, or social desirability bias. This concern may be particularly relevant in family-centered cultural contexts, where caregivers may feel pressure to report socially acceptable or family-consistent responses. Future studies should incorporate multi-source data, including clinician assessments, family meeting observations, resident clinical information, medical records, or qualitative interviews, to strengthen measurement validity and provide a more comprehensive understanding of caregiver decisional conflict.

Fourth, test–retest reliability was not assessed because the modified DCS was administered only once within a cross-sectional survey design. Therefore, although the scale demonstrated excellent internal consistency, the temporal stability of the modified DCS could not be established. Future longitudinal validation studies should administer the scale repeatedly over an appropriate interval to determine whether caregiver decisional conflict scores remain stable when the decision-making context has not substantially changed.

Fifth, although the modified DCS was modeled as a single latent construct, decisional conflict among surrogate family caregivers is conceptually multidimensional. In LTC settings, caregiver decisional conflict may involve cognitive uncertainty, emotional distress, ethical burden, family responsibility, relational pressure, and concern for the resident’s welfare. Therefore, the single-factor model should be understood as representing an overall decisional conflict construct rather than the full complexity of surrogate caregiving experiences. Future studies should examine multidimensional, hierarchical, and bifactor models of the modified DCS in larger samples to determine whether a strong general decisional conflict factor coexists with specific subdomains, such as information-related uncertainty, value clarification, perceived support, emotional burden, and family–professional communication.

Sixth, the modified DCS demonstrated excellent internal consistency, with Cronbach’s α = 0.98. However, this very high alpha value should be interpreted with caution, as it may indicate substantial conceptual overlap or redundancy among items rather than only strong reliability. Although the full 16-item scale was retained in this initial validation study to preserve conceptual coverage of decisional conflict, future studies should conduct additional item-level analyses, including corrected item-total correlations, inter-item correlations, item response theory analyses, and item reduction procedures. Such analyses would help determine whether a shorter version of the modified DCS could maintain adequate reliability, validity, and clinical interpretability while reducing respondent burden.

Seventh, criterion-related validity was not examined because external validation measures were not concurrently collected. Related constructs such as caregiver burden, anxiety, depressive symptoms, decision regret, decision self-efficacy, caregiver distress, and perceived quality of family–clinician communication were not included in the survey. Therefore, although the modified DCS demonstrated strong internal consistency and construct validity, its external validity in relation to these theoretically relevant outcomes remains to be established. Future studies should evaluate criterion-related validity by examining correlations between the modified DCS and caregiver-centered constructs, including caregiver burden, anxiety, decision regret, decision self-efficacy, and perceived decision quality.

Eighth, the present study was conducted in Taiwanese LTC settings, where caregiver decision-making may be shaped by culturally embedded factors such as filial responsibility, family-centered decision-making, family consensus, family hierarchy, and deference to healthcare professionals. However, these cultural factors were discussed as contextual background rather than directly operationalized or empirically measured in the present model. Therefore, the findings should not be interpreted as direct evidence of cultural mechanisms underlying caregiver decisional conflict. Future studies should directly measure cultural influences on surrogate decision-making by incorporating validated cultural constructs or developing context-specific measures of filial responsibility, family decision norms, perceived family obligation, family hierarchy, and professional authority. These variables may be examined as predictors, mediators, or moderators of decisional conflict.

Finally, several potentially important clinical, relational, and ethical determinants were not directly modeled, including resident clinical severity, frailty, comorbidity burden, cognitive impairment, prognosis, ethical burden, moral distress, family hierarchy, and clinician–caregiver power asymmetry. These factors may influence both the decision-making process, and the degree of decisional conflict experienced by caregivers. Future research should incorporate these clinical and contextual variables as covariates, moderators, or mediators. In addition, future studies should consider stratified sampling by decision urgency, prior SDM experience, caregiver relationship to the resident, and educational level, as these factors may affect decisional conflict and the applicability of the modified DCS across different caregiver groups.

## 5. Conclusions

In conclusion, the modified Decisional Conflict Scale demonstrated strong psychometric properties in this initial validation study and appears to be a reliable and valid instrument for assessing overall decisional conflict among primary caregivers involved in unplanned hospitalization decisions in Taiwanese long-term care settings. The findings suggest that caregiver readiness, information adequacy, perceived support, and communication processes are meaningfully associated with caregivers’ decisional experiences. However, given the cross-sectional design, these associations should not be interpreted as causal relationships. The findings should also be understood within the cultural and institutional context of Taiwanese long-term care, where family-centered decision-making norms, filial responsibility, and family–professional interactions may shape caregiver participation and decisional conflict.

Future studies should validate the modified scale in larger, independent, and more diverse samples, including long-term care settings across different cultural and healthcare systems. Further research should examine the test–retest reliability, temporal stability, criterion-related validity, responsiveness to decision-support or shared decision-making interventions, and performance of the modified DCS in multidimensional, hierarchical, or bifactor models that incorporate emotional, ethical, social, cultural, relational, and clinical factors. Although the modified DCS may serve as a potential assessment tool for identifying caregiver decisional conflict, its clinical use requires further evaluation within structured implementation pathways, such as family meetings, communication protocols, decision aids, and interdisciplinary care planning. Therefore, the present study provides initial psychometric evidence for the modified DCS, while broader clinical application and cross-cultural generalization require additional validation.

## Figures and Tables

**Table 1:** Demographic characteristics of primary caregivers with and without prior shared decision-making experience.

Characteristic	With Prior SDM Experience (*n* = 189)	Without Prior SDM Experience (*n* = 16)
**Gender**		
Men	84 (44.5)	7 (43.8)
Women	105 (55.5)	9 (56.2)
**Age group (years)**		
20–29	32 (16.9)	3 (18.8)
30–39	24 (12.7)	1 (6.3)
40–49	22 (11.6)	5 (31.3)
50–59	30 (15.9)	0 (0.0)
60–69	31 (16.4)	4 (25.0)
70–79	25 (13.2)	2 (12.5)
80–89	25 (13.3)	1 (6.1)
**Residents’ identity**		
Low income	35 (18.5)	4 (25.0)
Medium to low income	4 (2.1)	1 (6.2)
Emergency resettlement	96 (50.8)	8 (50.0)
Others	54 (28.6)	3 (18.8)
**Education level**		
High school or below	101 (53.5)	10 (43.8)
College/University	82 (43.4)	6 (56.2)
Graduate school	6 (3.1)	0 (0.0)
**Occupation**		
Yes	107 (56.6)	6 (37.5)
No	82 (43.4)	10 (62.5)

Note: Bold text indicates variable names or construct labels. Percentages may not total 100% due to rounding. To improve comparability, only demographic variables available for both groups are presented in the merged table.

**Table 2:** Reliability and validity estimate of the modified Decisional Conflict Scale.

Construct/Item	Standardized Factor Loading	Cronbach’s *α*	Composite Reliability (CR)	Average Variance Extracted (AVE)
Decisional Conflict		0.98	0.98	0.78
(R) B1. This decision comes naturally to me, as I have a strong sense of clarity and certainty.	0.85			
(R) B2. I possess a clear understanding of how to approach this decision.	0.85			
(R) B3. I am confident in my ability to determine what is best for my family.	0.83			
(R) B4. I am fully aware of all available options for my family in this decision.	0.86			
(R) B5. I feel well-informed about the advantages associated with each option.	0.90			
(R) B6. I have a comprehensive understanding of the drawbacks associated with each option.	0.90			
(R) B7. I recognize the importance of the benefits associated with this decision.	0.89			
(R) B8. I acknowledge the significance of the disadvantages in this decision.	0.90			
(R) B9. I am capable of prioritizing between the benefits and drawbacks of options.	0.89			
(R) B10. I feel liberated from external pressure while making this decision.	0.90			
(R) B11. I have ample support from others in my decision-making process.	0.90			
(R) B12. I have received sufficient advice to make an informed decision.	0.84			
(R) B13. I am confident that my decision is based on all relevant information.	0.88			
(R) B14. My decisions align with what matters most to my family.	0.89			
(R) B15. I have full confidence in my ability to maintain this decision.	0.90			
(R) B16. I feel a deep sense of contentment with my decision.	0.88			

Note: R = reverse-coded items; standardized loadings ≥ 0.70 indicate adequate factor reliability.

**Table 3:** Model fit indices for the confirmatory factor analysis (CFA).

Fit Index	Ideal Criteria	Observed Value	Interpretation
Bollen–Stine χ^2^	-	156.324	-
df	-	104	-
χ^2^/df	1–3	1.503	Good fit
GFI	>0.90	0.966	Excellent
AGFI	>0.90	0.961	Excellent
RMSEA	<0.08	0.050	Good
SRMR	<0.08	0.038	Excellent
TLI (NNFI)	>0.90	0.987	Excellent
CFI	>0.90	0.988	Excellent

Note: “-” indicates not applicable or not evaluated for the corresponding criterion. All fit indices fall within acceptable or optimal thresholds, indicating strong construct validity.

## Data Availability

The dataset generated and analyzed during the current study contains information derived from human participants and is subject to ethical and privacy restrictions. According to the requirements of the Taipei Medical University Joint Institutional Review Board (TMU-JIRB), the raw data cannot be made publicly available in order to protect participant confidentiality. However, data presented in this study can be made available from the corresponding author upon reasonable request.

## Supplementary Materials

The following supporting information can be downloaded at: https://www.mdpi.com/article/10.3390/healthcare14121754/s1, Table S1: Factor loadings for all modified DCS items (EFA and CFA); Table S2: CFA model fit indices for the modified DCS (*n* = 103); Table S3: SEM direct, indirect, and total effects (standardized); Table S4: Bootstrap mediation estimates (95% CI); Table S5: Proposed Short Form of the Modified DCS (example).
