# Supplementary material for: Modified Decisional Conflict Scale for Primary Caregivers in Long-Term Care Facilities: Psychometric Validation Using Structural Equation Modeling
Source: Healthcare (Basel). 2026 Jun 18;14(12):1754. doi: 10.3390/healthcare14121754 (PMC13299319; doi:10.3390/healthcare14121754)
Supplement: Supplementary file 1 [file healthcare-14-01754-s001.zip › healthcare-4278497-supplementary.pdf]

Table S1. Factor loadings for all modified DCS items (EFA and CFA)

Latent Construct	Item Code	Item Description	Source	Reverse-coded	EFA Factor Loading	CFA Factor Loading	*p*-Value
Decision Antecedents	DA1	Caregiver readiness for decision-making	Conceptually derived	No	–	Included in latent construct	–
DA2	DA2	Information adequacy prior to decision-making	Conceptually derived	No	–	Included in latent construct	–
DA3	DA3	Perceived decisional support from healthcare professionals and family members	Conceptually derived	No	–	Included in latent construct	–
Decision-Making Process	DP1	Communication quality during hospitalization discussions	Conceptually derived	No	–	Included in latent construct	–
DP2	DP2	Caregiver participation in shared decision-making	Conceptually derived	No	–	Included in latent construct	–
DP3	DP3	Collaborative discussion and value clarification	Conceptually derived	No	–	Included in latent construct	–
Decisional Conflict	B1	This decision comes naturally to me, as I have a strong sense of clarity and certainty.	Adapted from DCS	Yes	0.85	0.85	<0.001

Latent Construct	Item Code	Item Description	Source	Reverse-coded	EFA Factor Loading	CFA Factor Loading	*p*-Value
B2	B2	I know how to approach this decision effectively.	Adapted from DCS	Yes	0.85	0.85	<0.001
B3	B3	I am confident in my ability to determine what is best for my family.	Adapted from DCS	Yes	0.83	0.83	<0.001
…	…	…	…	…	…	…	…
B16	B16	I feel a deep sense of contentment with my decision.	Adapted from DCS	Yes	0.88	0.88	<0.001

Notes:

- EFA: Half-sample exploratory factor analysis (n = 102)
- CFA: Remaining half-sample confirmatory factor analysis (n = 103)
- Reverse-coded: "Yes" indicates reverse scoring for consistency
- Decision Antecedents and Decision-Making Process were latent constructs in SEM

Table S2. CFA model fit indices for the modified DCS (n = 103)

Model	χ^2 (df)	*p*-Value	CFI	TLI	RMSEA	SRMR
Single-factor CFA	113.27 (104)	0.25	0.99	0.98	0.03	0.04

Notes:

- CFA applied on half of the sample (n = 103) to confirm single-factor structure
- Robust Bollen–Stine bootstrap applied for non-normality

Table S3. SEM direct, indirect, and total effects (standardized)

Path	Direct Effect	Indirect Effect	Total Effect
Decision Antecedents → Decision-Making Process → Decisional Conflict	0.52	0.16	0.68
Decision Antecedents → Decisional Conflict	0.44	–	0.44

Notes:

- All effects standardized
- Indirect effects tested using bootstrap (*n* = 5000)

Table S4. Bootstrap mediation estimates (95% CI)

Mediation Path	Bootstrap Estimate	95% CI	*p*-Value
Decision Antecedents → Decision-Making Process → Decisional Conflict	0.16	0.08–0.26	<0.001

Notes:

- Bias-corrected bootstrap with 5000 resamples
- Confirms partial mediation of Decision-Making Process

Table S5. Proposed Short Form of the Modified DCS (example)

Item Code	Item Description	Reverse-coded	Notes
B2	I know how to approach this decision effectively.	Yes	Retained for clarity
B5	I feel well-informed about the advantages associated with each option.	Yes	Covers information adequacy
B11	I have ample support from others in my decision-making process.	Yes	Covers perceived support
B14	My decisions align with what matters most to my family.	Yes	Covers value clarity
B16	I feel a deep sense of contentment with my decision.	Yes	Captures overall decisional confidence

Notes:

- Short form designed for reduced respondent burden while retaining psychometric coverage
- Future validation required before clinical use
